# Captive-reared Delta Smelt (*Hypomesus transpacificus*) exhibit high survival in natural conditions using *in situ* enclosures

**DOI:** 10.1371/journal.pone.0286027

**Published:** 2023-05-26

**Authors:** Melinda R. Baerwald, Nicole Kwan, Catarina Pien, Grace Auringer, Evan W. Carson, Dennis E. Cocherell, Luke Ellison, Nann A. Fangue, Amanda J. Finger, Daphne A. Gille, Haley Hudson, Tien-Chieh Hung, Ted Sommer, Troy Stevenson, Brian M. Schreier

**Affiliations:** 1 California Department of Water Resources, West Sacramento, California, United States of America; 2 Department of Animal Science, University of California – Davis, Davis, California, United States of America; 3 San Francisco Bay-Delta Fish and Wildlife Office, U.S. Fish and Wildlife Service, Sacramento, California, United States of America; 4 Department of Wildlife, Fish and Conservation Biology, University of California – Davis, Davis, California, United States of America; 5 Department of Biological and Agricultural Engineering, University of California – Davis, Davis, California, United States of America; CIFRI: Central Inland Fisheries Research Institute, INDIA

## Abstract

Conservation of endangered fishes commonly includes captive breeding, applied research, and management. Since 1996, a captive breeding program has existed for the federally threatened and California endangered Delta Smelt *Hypomesus transpacificus*, an osmerid fish endemic to the upper San Francisco Estuary. Although this program serves as a captive refuge population, with experimental releases being initiated to supplement the wild population, it was uncertain how individuals would survive, feed, and maintain condition outside hatchery conditions. We evaluated this and the effects of three enclosure designs (41% open, 63% open, and 63% open with partial outer mesh wrap) on growth, survival, and feeding efficacy of cultured Delta Smelt at two locations (Sacramento River near Rio Vista, CA and in Sacramento River Deepwater Ship Channel) in the wild. Enclosures exposed fish to semi-natural conditions (ambient environmental fluctuations and wild food resources) but prevented escape and predation. After four weeks, survival was high for all enclosure types (94–100%) at both locations. The change in condition and weight was variable between sites, increasing at the first location but decreasing at the second location. Gut content analysis showed that fish consumed wild zooplankton that came into the enclosures. Cumulatively, results show that captive-reared Delta Smelt can survive and forage successfully when housed in enclosures under semi-natural conditions in the wild. When comparing enclosure types, we observed no significant difference in fish weight changes (*p* = 0.58–0.81 across sites). The success of housing captive-reared Delta Smelt in enclosures in the wild provides preliminary evidence that these fish may be suitable to supplement the wild population in the San Francisco Estuary. Furthermore, these enclosures are a new tool to test the efficacy of habitat management actions or to acclimate fish to wild conditions as a soft release strategy for recently initiated supplementation efforts.

## Introduction

Studying species near extinction is difficult yet often essential to support their recovery. Causes of species declines can be complex and challenging to disentangle if there are critical knowledge gaps about basic biology, ecology, and life history of the species [1]. Even when resources are available to conduct conservation and restoration actions, determining effectiveness and adaptively managing these actions can be difficult if species are at extremely low abundances [2]. Under these circumstances, use of conspecifics obtained from other sources (e.g., hatcheries) are invaluable, and sometimes the only option for informing management actions and supporting recovery of the wild population. However, introducing captive-reared fish into the wild presents numerous challenges, even if only done on a limited research scale [3]. One of these challenges is determining the best way to release fish, such as with acclimatization near the release site (soft release) versus without it (hard release), to maximize subsequent survival [4].

In the San Francisco Estuary (Estuary), the Delta Smelt *Hypomesus transpacificus*, an endemic pelagic osmerid fish species, has precipitously declined in abundance in recent decades. The species is currently listed as endangered under the California Endangered Species Act (ESA) and as threatened (but meriting reclassification to endangered) under the federal ESA [5,6]. Concerns regarding species extinction prompted the establishment of a captive breeding program in 1996 and a genetically managed refuge population in 2008 at the University of California–Davis Fish Conservation and Culture Laboratory (FCCL; Byron, CA, USA; [7]). The species has received national attention due to the ecological and socioeconomic effects of its management [8,9] with its decline attributed to a suite of ecosystem changes, including water diversions, contaminants, habitat loss, and introduced species [10–12]. Despite ongoing and proposed restoration efforts and management actions [13–15], the ecological resilience and threat of extinction in the wild [16] are concerning as species abundance is at or below the detection limits of the monitoring programs used to assess its status [17]. Hence, there is growing consensus that captive-reared Delta Smelt are likely to play an important role in recovery of this species through future supplementation and field experimentation [18]. Proper tools are necessary to evaluate the effectiveness of a supplementation program or management actions intended to benefit the species.

*In situ* use of captive-reared Delta Smelt in enclosures may be a valuable tool for research and conservation efforts (including as a soft release strategy for supplementation) but are only likely to succeed if the unique needs of Delta Smelt are considered throughout the deployment period. Extensive research has found Delta Smelt are sensitive to environmental changes (e.g., increasing temperatures [19]) and typically do not survive collection from the field unless particular nets (e.g., lampara) and handling procedures [20]are used to limit capture, handling, and transport stress [21]. In captivity, the species requires intensive aquaculture support to complete its life cycle [20]. Recently, enclosures were designed specifically for juvenile to adult Delta Smelt (Gille et al., unpublished work), with design considerations given to factors that may affect their survival, growth, and ability to feed. However, prior to the study detailed herein, the enclosures were not tested under field conditions in the Estuary with variable and episodic heavy flows. Given the highly sensitive nature of the species, and to increase the likelihood of study success, we decided to test multiple enclosure types to achieve optimal rearing under wild conditions as a form of bet-hedging.

**Fig 1:**
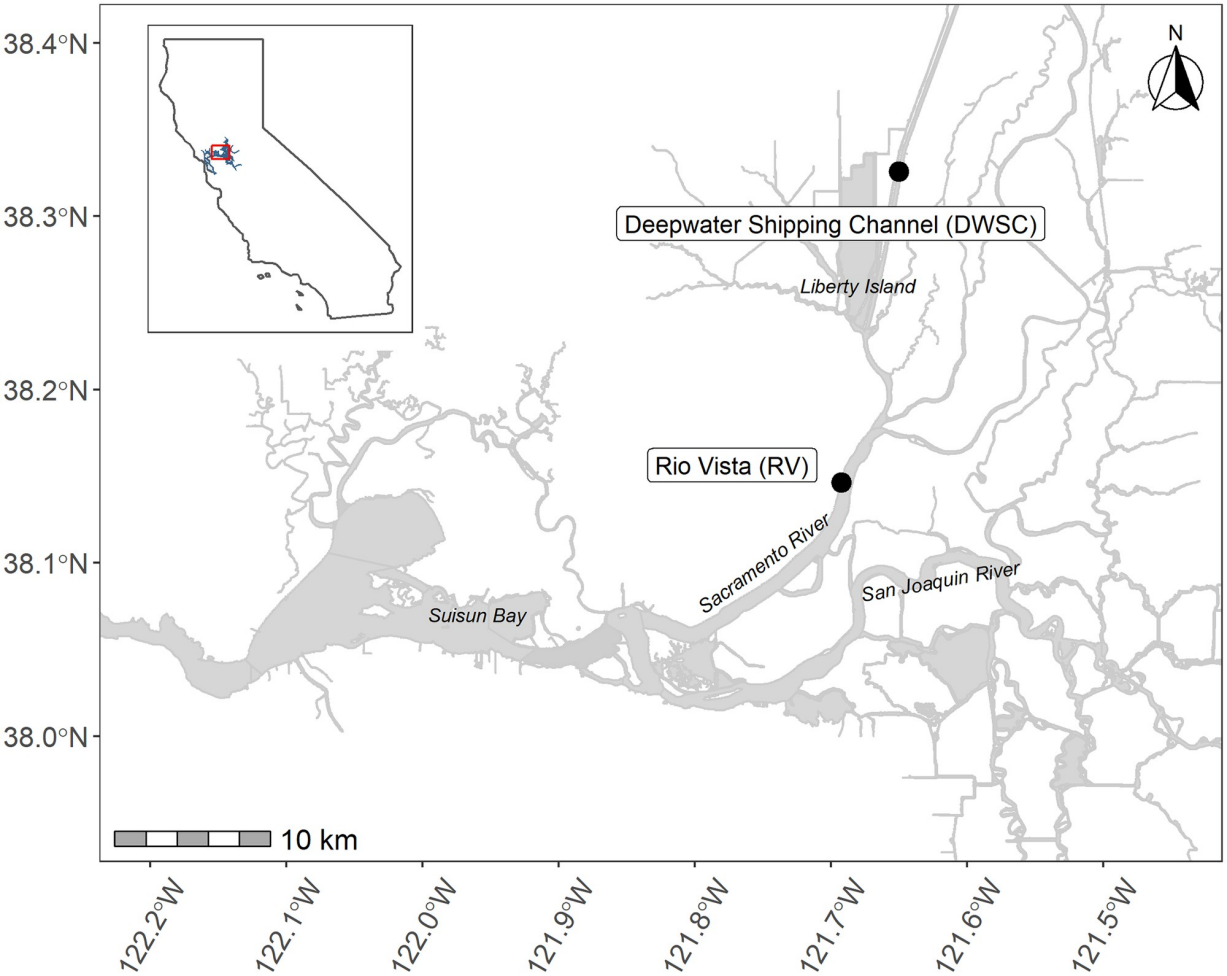
Map of Delta Smelt enclosure study sites. The map was created using the sf package [26,27] in R [28] with waterways [29] and California [30] shapefiles.

To test the potential use of new enclosures specifically designed for juvenile to adult Delta Smelt, we conducted field trials with captive-reared Delta Smelt at two sites in the Estuary, which are in core Delta Smelt habitat (Fig 1; [22]), over a one-month period. We hypothesized that: 1) captive-reared Delta Smelt would have different growth and survival across enclosure types, but overall survival at the end of the four-week deployment period would be low due to the sensitivity of the species to handling and stress [23]; and 2) Delta Smelt foraging in the enclosures would vary based on enclosure type and the relative proportions of prey taxa available in the environment. The intent of this study was to inform the conservation of Delta Smelt, and by extension other captively-reared threatened fishes, through the development and application of a field tool that could enable testing of management actions, including supplementation [24] and habitat improvements such as wetland restoration [25] and marsh salinity control [15].

## Materials and methods

The study protocol was reviewed and approved by the UC Davis IACUC (Protocol #19841). MS-222 was used for anesthesia and euthanasia. Study activities were authorized by a US Fish and Wildlife Service 10(a)(1)(A) permit (Permit: TE-027742) and a California Department of Fish and Wildlife Memorandum of Understanding (2081a-2018-0007-R3).

### Experimental and enclosure design

In winter and spring of 2019, we deployed captive-reared Delta Smelt in enclosures for four-weeks at two tidal sites in the upper Estuary: in the Sacramento River near the city of Rio Vista (RV) and in the Sacramento Deepwater Shipping Channel (DWSC), 10.5 km upstream of the confluence with Cache Slough (Fig 1). We selected these periods and sites to coincide with the timing and locations in which monitoring programs have caught wild Delta Smelt in recent years [31].

**Fig 2:**
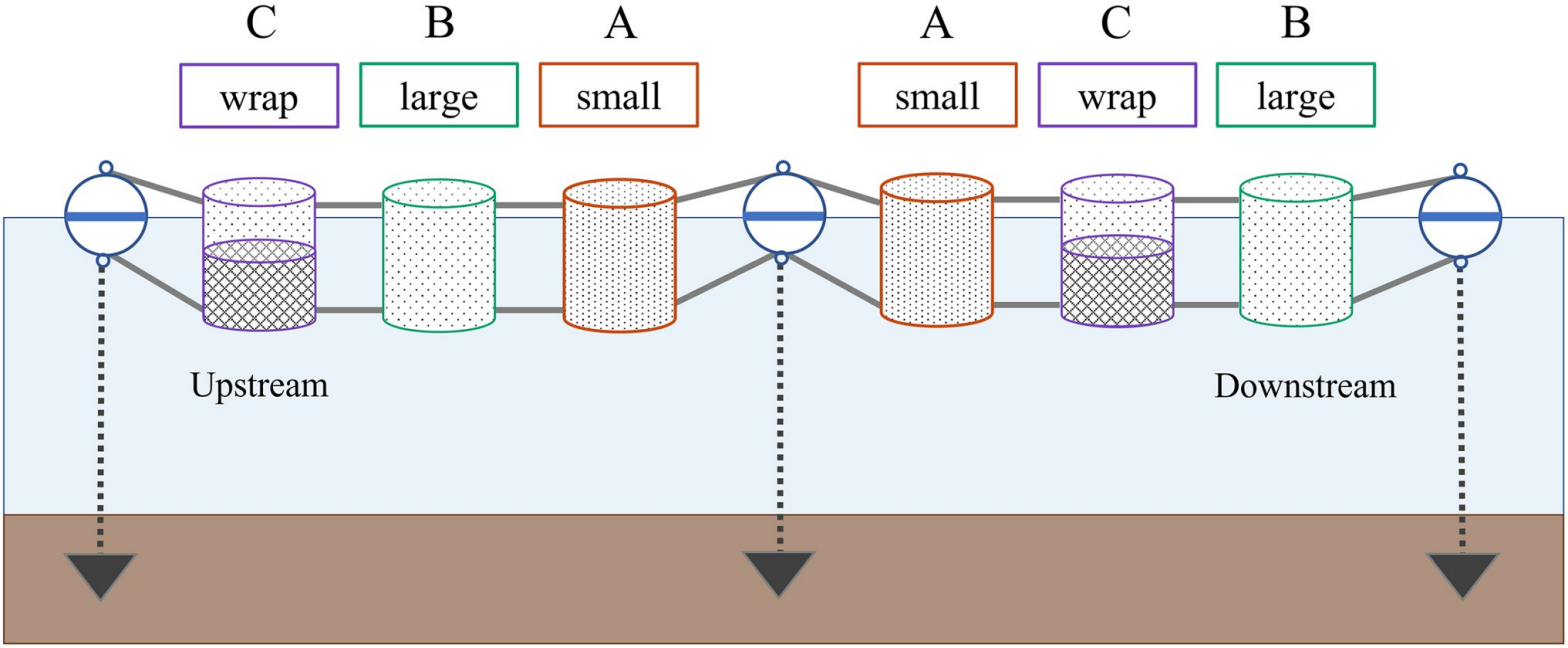
Experimental design. Schematic of an enclosure deployment showing layout of enclosure types (A—small, B—large, or C—wrap) and the anchoring system. The layout was kept the same at both study sites.

For our field trials, we constructed and deployed three enclosure types (Fig 2) to determine if their unique design aspects would influence the growth, survival and feeding efficacy of Delta Smelt. Prior to field deployment, enclosures were designed, tested, and constructed, incorporating key aspects from Delta Smelt physiology and behavior into the 1.0 m in diameter and 1.3 m tall cylindrical design (Gille et al., unpublished work). The material types that performed best in laboratory trials were woven stainless steel wire mesh (60% openness) and perforated steel (40% openness). These material types allowed a moderate amount of flow, and thus Delta Smelt prey items, to enter the enclosures but were protective against higher flows that could cause impingement and prevented escape or exposure to predators. Therefore, we wanted to construct enclosures with similar openness for testing under field conditions and used perforated stainless steel with 41% and 63% openness for our first and second enclosure design types, respectively. Additionally, we constructed a third enclosure type with an external wrap of woven mesh on the lower half to provide further velocity reduction since Delta Smelt are not consistently strong swimmers [32]. These different material perforation sizes were chosen to find a functional balance between the need for water velocity reduction (less perforation, putatively a velocity refuge for the fish) and ability for ambient food to enter the enclosures (larger perforations preferred for zooplankton passage). We deployed these three enclosure variations (designated as enclosure design A, B, and C), each defined by the type and percent openness of the side and bottom perforated material (Table 1).

**Table 1:** Enclosure design specifications and materials.

Enclosure design type	Side/bottom material	Hole Size (mm)	Center to Center Distance (mm)	Openness (%)	Mesh Size of wrap (mm)
**A**	perforated steel sheet	3.18	4.76	41	N/A
**B**	perforated steel sheet	3.97	4.76	63	N/A
**C**	perforated steel sheet with outer wrap of stainless steel mesh on lower 50%	3.97	4.76	63 + mesh	4 x 4

We deployed the enclosures in two replicate sets of three (A, B, and C; six total enclosures) with each set attached together in a line, parallel to shore, using 3 m of 6.35 mm stainless steel cable encased in 25.4 mm diameter PVC pipe to add rigidity and maintain distance between each enclosure (Fig 2). Each set was attached at both ends to an anchor buoy with 109 kg buoyancy and held in place by a 61 kg pyramid anchor buried in the substrate. The resulting system was able to move vertically with the tides while maintaining the same relative distance between enclosures.

### Fish acclimation and deployment

We obtained captive-reared, adult Delta Smelt from the FCCL (UC Davis IACUC protocol #19841). Fish were acclimated in the hatchery to more closely mimic water quality, food, and flow conditions they may experience in the wild prior to being transferred to the field. Due to limited tank space, fish for the RV and DWSC deployments were acclimated concurrently despite being moved to the field at different times. At 206 days post hatch (dph; approximately one month prior to the first field deployment), fish were transitioned from temperature-controlled water (16°C) to raw ambient water (11.5–12.0°C), and at 207–210 dph, they were transitioned from dry pelletized food (Biovita Starter #1, Bio-Oregon, Longview, WA) to live, unenriched, newly hatched brine shrimp nauplii (*Artemia franciscana*, INVE Aquaculture Inc, Salt Lake City, UT). Subsequently at 223–224 dph, flow in the acclimation tanks was increased, starting with one day at 0.15 m/s, and the following days at 0.30 m/s. At 207 dph, we tagged all fish with Visible Implant Alphanumeric (VIA) tags (Northwest Marine Technology Inc, Olympia, WA; [33]) for individual identification and weighed (grams, g), and measured (fork length, mm). For both tagging and swabbing, we anesthetized the fish using buffered Tricaine Methanesulfonate (MS-222, 0.1 g/L). We transported 384 Delta Smelt to RV on January 23^rd^ (243 dph; 46 to 72 mm fork length) and 360 fish to the DWSC on February 27^th^ (278 dph; 48 to 75 mm fork length) in insulated, black 19 L buckets with screw-top lids at a density of 34 fish (RV) and 30 fish (DWSC) per bucket (1.8 and 1.6 fish/L, respectively). Buckets were filled with ambient water and salted to 5 ppt salinity to minimize stress and supersaturated with pure oxygen [20]. Upon arrival at each site, we transferred buckets to a boat and then to the enclosures. We emptied two buckets into each enclosure using a water-to-water transfer after allowing for one minute of water exchange between the buckets and the ambient water. This led to densities of 64 fish/enclosure at RV and 60 fish/enclosure at the DWSC (approximately 0.067 and 0.059 fish/L). Informed by lab-based studies, we selected these densities to allow enough fish for natural shoaling behaviors, which reduces stress [34], while not detrimentally limiting food availability. To buffer against expected transportation-induced mortality, we included extra fish (*n* = 4 per enclosure) during our first deployment at the RV site. As there was no evidence of transportation-induced mortality, we did not include additional fish for the subsequent DWSC deployment (i.e., *n* = 60 fish/enclosure).

### Field methods

After transferring the fish, we checked enclosures each weekday for damage and biofouling as well as any surface mortalities, which were collected, measured (fork length, mm), and stored in individual WhirlPacks on ice prior to transfer to a -20°C freezer. For the first two weeks of the experiment at RV, we swept a net along the bottom of each enclosure in an attempt recover mortalities; however, we then suspended this practice because no mortalities were recovered, and we were concerned it might cause unnecessary stress for the other fish.

During daily checks, we used a SEA-GEAR conical 0.5 m x 2 m plankton net with 53 μm mesh and a General Oceanics flowmeter (Model 2030R) suspended from the center of the net mouth to collect zooplankton samples adjacent to the enclosures. We towed the net below the surface of the water for two minutes. For some dates, zooplankton tows were not included due to high wind conditions (*n* = 2 out of 9 samples at each site). We stored all samples in 1 L wide-mouth Nalgene bottles and preserved samples in 5% formalin dyed with Rose Bengal. We sent samples to BSA Environmental Services, Inc. (Beachwood, OH USA) for enumeration and identification to genus for cladocerans, order for harpacticoids, and species and life stage for calanoid and cyclopoid copepods.

During the daily checks, we collected physical water quality and velocity data. We used a YSI Pro DSS to collect measurements of water temperature (°C), dissolved oxygen (mg/L), specific conductivity (μSiemens/cm), electric conductivity (μSiemens/cm), pH, and turbidity (FNU), a Secchi disc to collected Secchi depth (m), and a portable handheld velocity meter (Hach, model #FH950) to collect three replicate velocity samples (m^3^/s; sensor positioned in the direction of greatest velocity), which were averaged over three 10-second readings.

### Fish dissections and measurements

At the conclusion of each one-month experimental period, we collected all fish from each enclosure. To access the fish, we lifted enclosures from the water using a boat-mounted hydraulic winch and lowered them into a small pool on the boat deck with approximately 15 cm of standing water. We then netted fish through the lid opening, transferring them to 19 L filled black buckets. We euthanized the fish with a lethal dose of buffered MS-222 (400 mg/L), after which they were weighed (g), measured (mm), and preserved in 10% formalin.

To assess diet, we dissected 10 fish per enclosure (60 fish per site). We grouped fish into size bins, and randomly selected 2–3 fish from each bin and removed the stomach for diet analysis at the University of Washington’s Wetland Ecosystem Team laboratory (WET lab; Seattle, WA USA). The total contents of each stomach were weighed and enumerated, and prey items were identified to the same taxonomic level as the zooplankton tow samples.

### Calculations and statistical analyses

In addition to collecting information on weight and length, we also calculated Fulton’s Condition Factor (K) to provide a metric of general health of each fish.

$$K=\frac{Weight}{Fork\;Length^{3\;}}x100$$

where weight is in grams and fork length is in centimeters. For analyses of how growth differed between cages, we chose weight as our key metric and calculated proportional change in weight: (**Weight**_**post-deployment**_** − Weight**_**pre-deployment**_**)/Weight**_**pre-deployment**_. While we used VIA tags to keep track of individual fish, a proportion of fish shed their tags, or may have been misread, leading to some mismatch between pre- and post-deployment fish. Tag loss/misread rates ranged from 4.8–8.4% across enclosure types and sites (*n* = 118–128) and did not appear to exhibit a pattern across site or enclosure type. Greater than 90% of fish were retained for analyses.

We conducted statistical analyses in R Version 4.0.5 [28]. To assess how fish growth and condition changed during the deployment, we conducted t-tests comparing pre-deployment fork lengths, weights and K with post-deployment weights and K. Weights and K were log-transformed. Due to the more nominal change associated with fork length, we used weight as the metric for assessing how growth varied by enclosure type. Enclosures were compared within but not across location, due to differences in initial age, length, and weight of the fish deployed at RV versus the DWSC. For RV, we tested a linear mixed-effects model (lmer) with the replicate enclosure (*n* = 2 per enclosure type; individual fish as samples) as a random effect (lmerTest package; [35]). Because enclosure type did not affect the variance for DWSC (variance = 0), we ran a simple linear model for DWSC. We then ran an analysis of variance (ANOVA) of each model fit to provide a type II model summary by the Satterthwaite method for degrees of freedom (lmerTest package for RV; [24] car package for DWSC; [36]). We used visual observations of residuals to evaluate assumptions of homoscedasticity and normality. While there was some deviation from normality at DWSC, given the sample size was large, and ANOVA is recognized to be robust to non-normal data [37], we determined this approach was preferable to non-parametric rank tests.

We did not perform statistical analyses on survival rates due to high survival rates across enclosures. Instead, we provided ranges for survival across the sampling sites and enclosure types.

To evaluate potential differences in foraging success across enclosure types, we analyzed instantaneous ration (IR) for ten fish per enclosure at each site. IR is a measurement of foraging performance calculated as a ratio of stomach content weight to body weight [38]. We modeled the log-transformed response variable IR, with enclosure type as the fixed effect and analyzed the model using ANOVA. We explored adding individual enclosures as a random effect; however, as the variance across enclosure types was zero, we did not include this term in the final model. For both this and the zooplankton model, we performed a Tukey HSD post-hoc test when model results were *P < 0*.*05* to determine which enclosure types differed. To test for normality and homoscedasticity of the model residuals, we employed the Shapiro-Wilk and Levene tests, respectively.

To visualize the relative abundance of different taxa among enclosure types, we calculated taxa proportion (taxa abundance/total abundance) for each fish’s diet and then averaged these taxa proportions across individuals from each enclosure type. Means were then fit to a 100% scale to estimate the mean percent contribution of each taxa to overall enclosure type diets. Averaging proportions instead of abundance across fish allowed for the diets of different sized fish to contribute equally.

To assess variation in food availability, we used a linear model to analyze mean zooplankton biomass across time and between the two sites using two-way repeated measures ANOVA. At each site, we averaged zooplankton biomass by week, then modeled zooplankton biomass as the response variable, with site and sampling week factors modeled as the fixed variables. We removed microzooplankton prior to analysis as they were caught in high abundance but are not commonly eaten by adult Delta Smelt during early spring, when other larger and more nutritious prey are available [17,39].

## Results

### Growth, condition and survival

**Fig 3:**
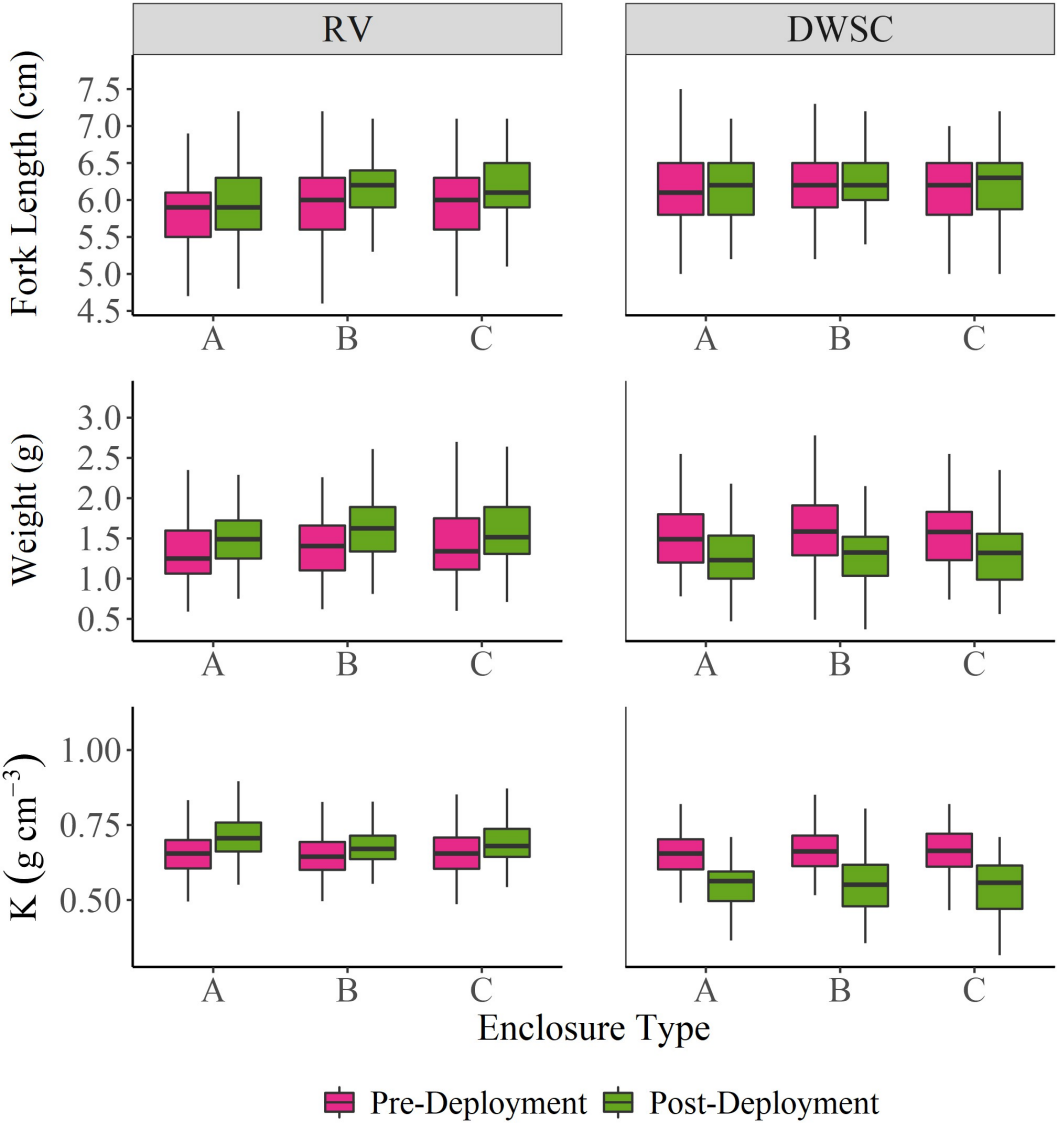
Boxplots of pre- and post-deployment smelt weight (g), fork length (cm), and K (condition factor; g cm^-3^) by enclosure type in Rio Vista (RV) and the Deepwater Shipping Channel (DWSC). A = small mesh, B = large mesh, C = large mesh with wrap.

**Fig 4:**
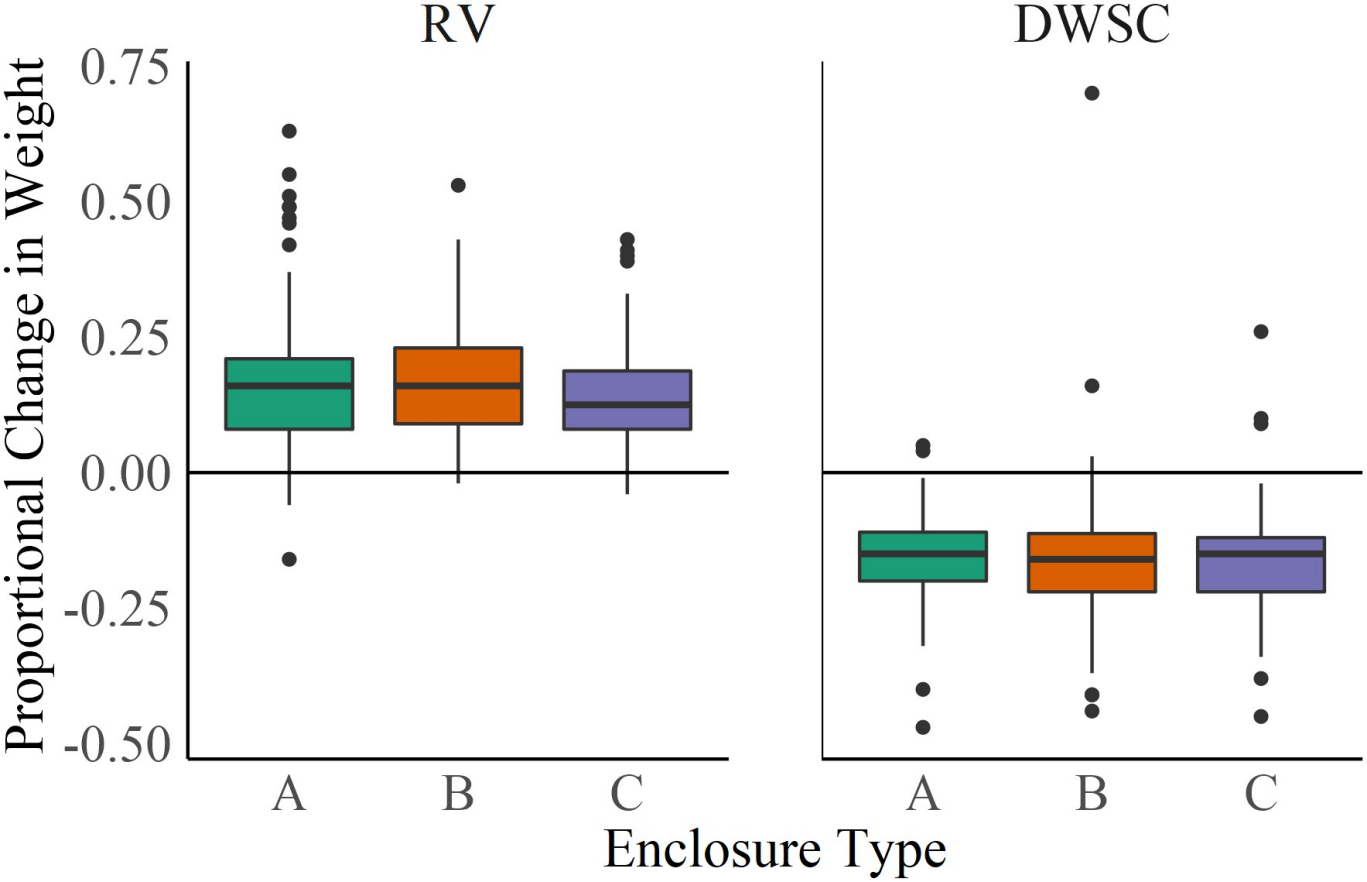
Boxplot of Delta Smelt proportional change in weight (g) by enclosure type in Rio Vista (RV) and the Deepwater Shipping Channel (DWSC). Horizontal lines indicate no change in weight. A = small mesh, B = large mesh, C = large mesh with wrap.

To assess smelt growth, we measured FL, weight, and K for each fish before and after deployment in the field (Fig 3). At RV, weight, fork length and K all increased over the duration of the study. Mean ± SE change in weight was 0.197 ± 0.006 g, and pre- and post-weight were significantly different from each other (t-test t = -34.791, df = 597.72, *p* < 2.2e-16), with mean percent change = +15.9% (Figs 3 and 4). Mean ± SE change in fork length was 0.164 ± 0.011 cm, and pre- and post-fork length were significantly different from each other (t-test t = -4.5123, df = 684.31, *p* = 7.547e-06), with mean percent change = +2.9% (Fig 3). Similarly, K increased (t-test t = -6.8555, df = 687.21, *p* = 1.6e-11; Fig 3), with mean ± SE change in K of 0.040 ± 0.004 g. Meanwhile, at the DWSC, fork length did not change, and weight and K decreased over the duration of the study. Mean ± SE change in weight was -0.248 ± 0.009 g and pre- and post-K were significantly different from each other (t = -14.814, df = 493.77, *p* < 2.2e-16), with mean percent change = -15.9%; Figs 3 and 4). Mean ± SE change in K was -0.111 ± 0.004, with significantly different pre- and post-K (t-test t = 11.802, df = 366.76, *p* < 2.2e-16; Fig 3). These analyses do not account for deceased fish that could not be measured due to degradation in the enclosure. For both RV (Table 2; ANOVA F = 0.2232, df = 2, *p* = 0.8122) and DWSC (ANOVA F = 0.5411, df = 2, *p* = 0.583), there was no significant effect of enclosure type on change in weight (Fig 4).

**Table 2:** Summary of mixed model results for Rio Vista deployment.

Fixed Effects	Predictors	Estimates	df	t value	CI	*p*
	(Intercept)	0.15	3.01	6.081	0.10–0.20	<0.001
	Mesh: Small	-0.02	2.98	-0.647	-0.09–0.05	0.518
	Mesh: Wrap	-0.01	2.99	-0.182	-0.08–0.06	0.855
**Random Effects (*N* = 345, enclosures = 6)**	**Groups**	**Variance**				
	Enclosure	0.033				
	Residual	0.091				

Model: log(proportional change in weight + 1) ~ Mesh + (1|Cage), REML = TRUE).

Delta Smelt survival for individual enclosures at RV ranged from 87.5% to 100.0%, with mean ± SE of 96.4% ± 0.8%. At the DWSC, survival ranged from 96.7% to 100.0%, with mean ± standard deviation of 98.6% ± 0.3%. Survival was similar among all enclosure types at both locations, with just one individual enclosure experiencing survival below 90% (Table 3).

**Table 3:** Summary of Delta Smelt survival by enclosure type for Rio Vista (RV) and Deepwater Shipping Channel (DWSC) deployments.

Site	Enclosure Type	Survival per Replicate	Mean Survival
RV	A	100%	100%
RV	B	88–100%	94%
RV	C	94–97%	95%
DWSC	A	97–100%	98%
DWSC	B	97–100%	98%
DWSC	C	98–100%	99%

For RV, *n =* 128 fish for each enclosure type. For DWSC, *n* = 120 fish for each enclosure type. There were 2 cages for each enclosure type.

### Diet

The effect of enclosure type on ration varied by site. Instantaneous ration (IR) differed across enclosure types at RV (ANOVA, *F*_2, 57_ = 4.25, *p* = 0.019) but not at the DWSC (ANOVA, *F*_2, 57_ = 2.53, *p* = 0.089). Mean IR at RV was higher in design B (0.25 ± 0.02 SE) than in design A (0.16 ± 0.02), with design C (0.23 ± 0.02) intermediate (Tukey HSD test, *p <* 0.05). There was no significant difference (Tukey HSD test, *p >* 0.05) in IR between enclosure types at the DWSC, where IR was highest in design B (0.22 ± 0.02) and near similar in design A (0.16 ± 0.02) and design C (0.16 ± 0.02).

**Fig 5:**
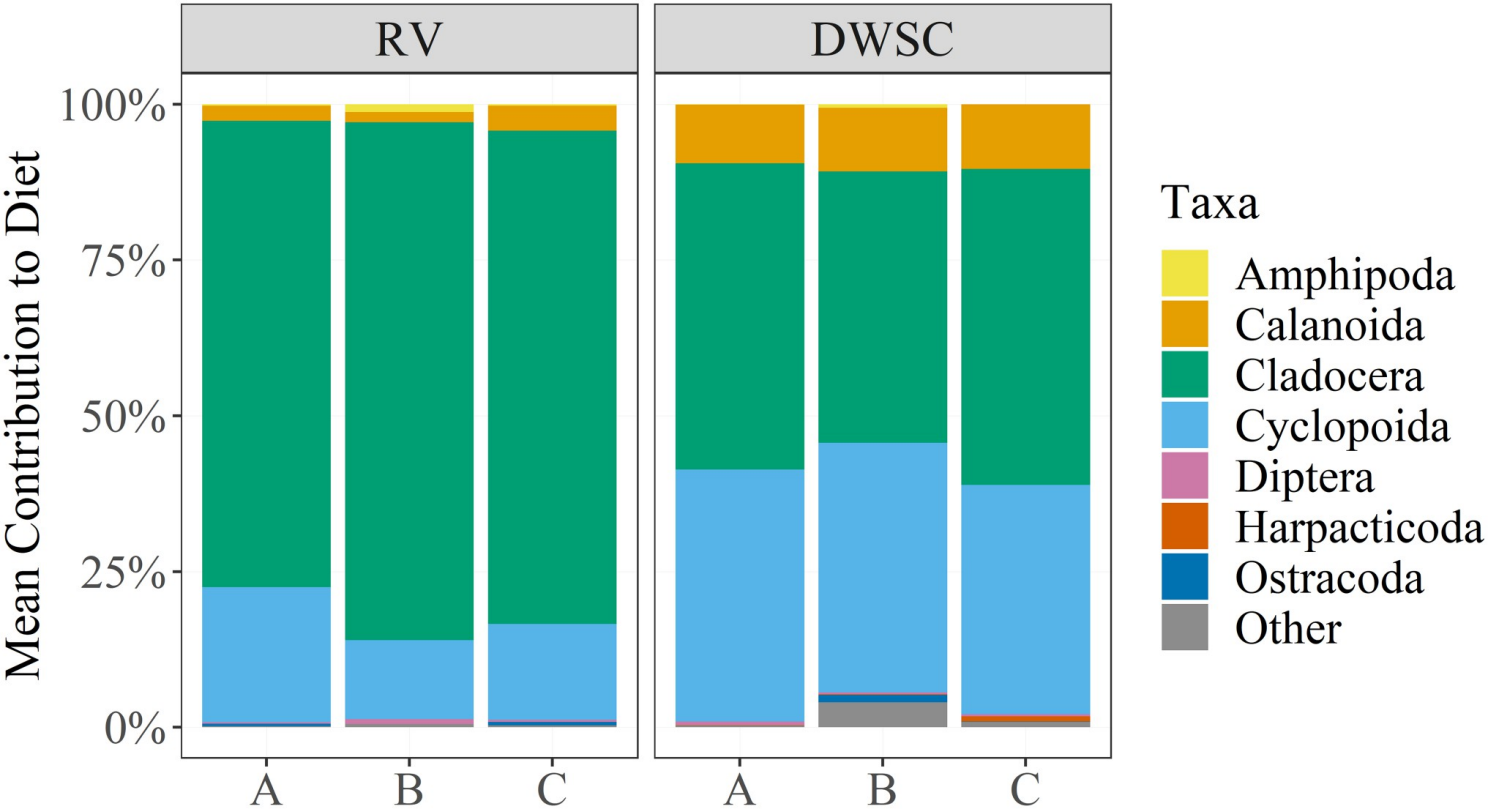
Proportion of prey consumed across enclosure types at each site (RV or DWSC). Data reflects abundance (count) of individuals consumed. Each bar represents the diet for *n* = 20 fish.

Delta Smelt diet composition also differed between sites, though variability among enclosure types was negligible (Fig 5). At RV, fish in the final days of the study mostly consumed cladocerans (79.1%) and cyclopoids (16.6%). The most abundantly consumed cladoceran was *Daphnia*, and the most abundantly consumed cyclopoid was *Acanthocylops sp*. (S1 Table). At the DWSC, fish mostly consumed cladocerans (47.8%), cyclopoids (39.1%), and some calanoids (10.0%). The most abundantly consumed cladoceran was *Chydorus sp*., and the most abundant cyclopoid consumed was *Acanthocyclops sp*. (S1 Table). Notably, the fish at RV and the DWSC were different ages and the diet results reported here only represent what fish were eating in the final days of the study. Analysis of taxa consumed and ration throughout the study may have produced different results.

### Zooplankton

**Fig 6:**
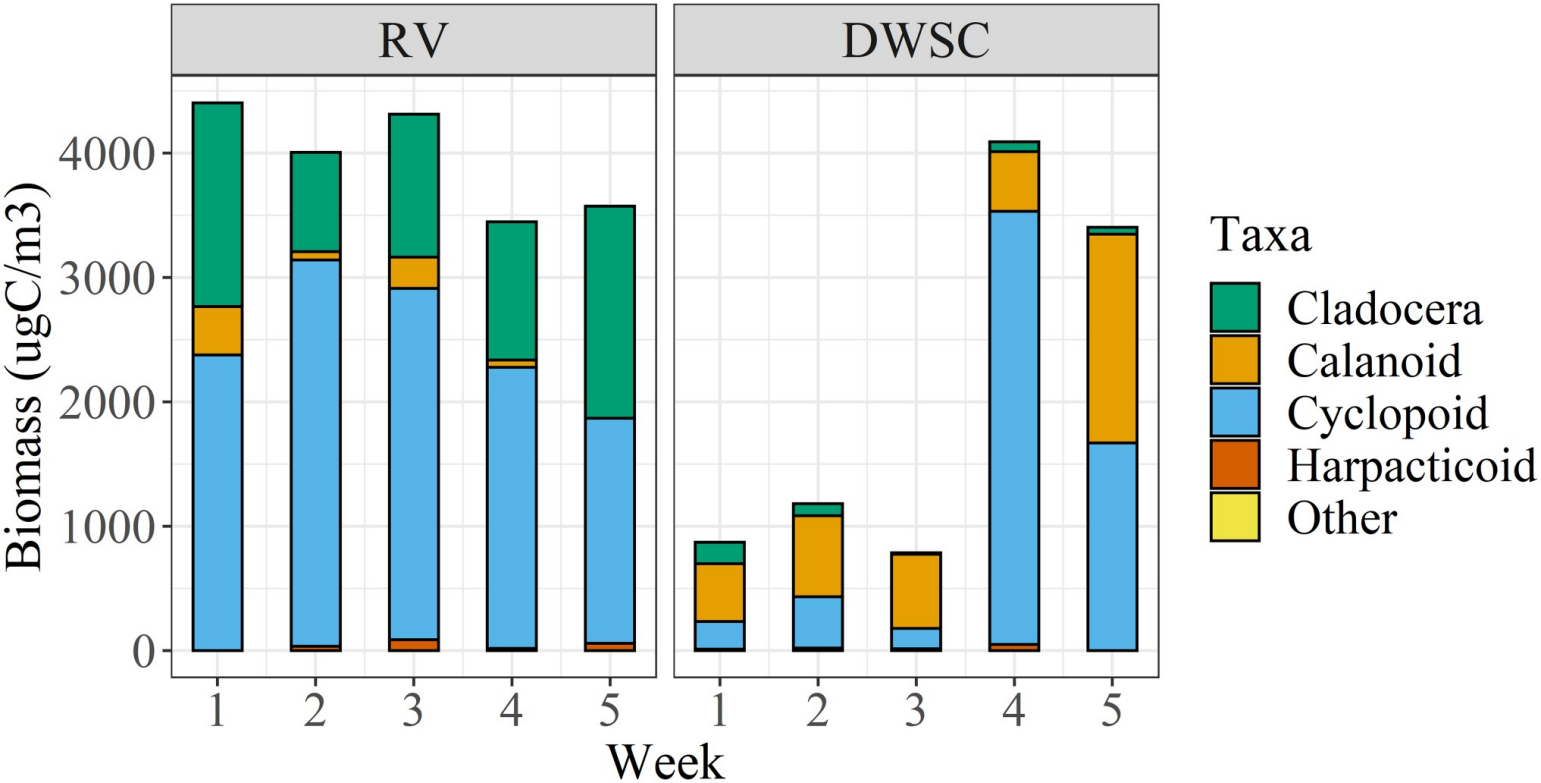
Zooplankton biomass, excluding microzooplankton, per week at Rio Vista (RV) and Deepwater Shipping Channel (DWSC). Note that “week” on the x-axis represents the first through final week of each deployment and that tows at the RV and the DWSC were collected during different time periods. Weeks 2, 3, 4 at RV and weeks 1 and 2 at the DWSC represent two zooplankton samples in the given week, while remaining samples represent 1 sample.

Zooplankton biomass (μgC/m^3^) varied by site (Fig 6). There was a significant difference between biomass at RV and the DWSC (Fig 6; ANOVA, *F*_1, 6_ = 14.67, *p* = 0.009), with higher zooplankton biomass at RV (Tukey HSD test, *p <* 0.05). There was no effect of sampling week on biomass (ANOVA, *F*_1, 6_ = 1.10, *p* = 0.334) but there was a significant interaction between sampling week and site as biomass stayed relatively stable across weeks RV and increased during the later weeks at DWSC (ANOVA, *F*_1, 6_ = 2.74, *p* = 0.026). Cyclopoid copepodites and *Daphnia* contributed the most to biomass at RV while calanoid and cyclopoid copepodites and *Sinocalanus doerrii* comprised most of the biomass at the DWSC (Fig 6, S2 Table). These results suggest that food was more abundant during the RV deployment than during the DWSC deployment.

### Water quality

Based on averages from discrete sampling events (Table 4), turbidity was higher at RV, resulting from concurrent extensive upstream flooding which exported sediment into the Sacramento River. Water temperature was higher at the DWSC, presumably because the DWSC deployment period spanned the warming spring months. Conductivity, dissolved oxygen, and pH were also higher at the DWSC than RV. Water velocity was higher at RV compared with the DWSC, due to its riverine location and stronger tidal influence. Comparisons of velocity measurements inside and outside of the enclosures at both locations indicated that enclosure design B blocked an average of 43.98% of flow, enclosure design C blocked 50.47%, and enclosure design A blocked 70.68%.

**Table 4:** Summary of discrete water quality data (minimum, mean, and maximum) at Rio Vista (RV) and the Deepwater Shipping Channel (DWSC) enclosure deployment locations.

Water quality metric	RV min	RV mean	RV max	DWSC min	DWSC mean	DWSC max
Secchi disc depth (m)	0.13	0.31	0.68	0.20	0.33	0.47
Water temperature (°C)	8.40	10.13	11.80	9.70	11.85	14.10
Dissolved oxygen (mg/L)	8.45	9.54	10.90	9.61	10.26	10.59
Specific conductivity (μS/cm)	127.00	213.95	283.00	366.00	528.07	695.00
Electrical conductivity (μS/cm)	87.00	153.86	209.00	259.00	396.93	538.00
pH	7.37	7.73	8.21	7.18	8.01	8.76
Turbidity (FNU)	13.90	46.59	99.63	17.60	27.11	38.23
Velocity (m/s)	0.001	0.015	0.029	0.002	0.011	0.020

Mean values are an average of measurements collected across each 4-week deployment.

## Discussion

This study demonstrates that captive-reared adult Delta Smelt can survive, feed, grow, and maintain good condition in enclosures kept in the wild for at least four weeks. The purpose of developing enclosures for captive-reared Delta Smelt was two-fold. First, they enable us to directly assess how Delta Smelt kept in enclosures respond to different environmental conditions in the wild (e.g., restored wetland habitat [13] or managed flow actions [15]) without the need to collect increasingly rare wild Delta Smelt. Second, experimentation using enclosures fills knowledge gaps about the condition and survival of captive-reared Delta Smelt in more natural conditions to inform release strategies for upcoming mandated supplementation efforts [40]. Captive breeding is a widely used conservation and management tool to support recovery programs for threatened fish species [41]. Although a captive breeding and augmentation program is often not recommended as a sole conservation strategy due to concerns of domestication selection [42] and the need for continual supplementation, it can be invaluable for allowing a species to persist while other conservation measures are being put into effect. For example, there is a captive breeding and augmentation program for the federally endangered Rio Grande Silvery Minnow *Hybognathus amarus*, which is considered necessary to avoid extinction until habitat restoration efforts take effect [43]. Salmonid species are frequently the focus of breeding and augmentation programs and some, such as winter-run Chinook *Oncorhynchus tshawytscha* and Coho *O*. *kisutch* salmon broodstock programs in California, use conservation hatchery best practices in attempt to build self-sustaining populations in the wild [44,45].

Prior to this study, it was unknown if captive-reared Delta Smelt could survive in the Estuary due to domestication effects in the hatchery [46] and fundamental shifts in the ecosystem that contributed to the decline of the species [12]. Our hypotheses of variable survival and fish condition across enclosure types as well as overall low survival due to the sensitive nature of the species was not observed. The success of the captive-reared fish and experimental enclosures measured as Delta Smelt survival (88–100% across enclosure types and locations), evidence of consumed zooplankton, and the positive change in weight and K at RV while in enclosures indicate that these fish can survive, feed, and maintain good condition in enclosures deployed in natural conditions. However, it is unknown if survival and K of captive-reared fish in enclosures translates to long-term survival under completely wild conditions, including interactions with competitors, predators, variable flows, and the opportunity to move into other habitats. It has been well-documented that captive-born fish reared in conventional hatcheries often exhibit altered behaviors when released into the wild, and these behaviors can potentially reduce their ability to successfully forage and escape predators [47–49]. It is unclear how relevant these studies, which examine fishes with very different life histories, are for Delta Smelt. To account for potentially altered behavior in preparation for supplementation, the FCCL practices some enrichment strategies to partially ameliorate maladaptive behaviors in the wild (e.g., low rearing densities, fed live prey at some life stages) and other strategies are being considered for future experimentation (e.g., introducing predator cues). Additionally, acclimating in the predator-free enclosures may enable captive-reared Delta Smelt to recover from transport stress and begin feeding prior to supplementation releases. A study using captive-reared Common Snook *Centropomus undecimalis* found that fish acclimated in enclosures for three days prior to release in an estuary had a mean recapture rate that was nearly two times higher than those that were directly released without acclimation [50].

As our primary study goals were to determine if captive-reared fish could survive in semi-wild conditions and to compare among the different enclosure types, we did not conduct statistical comparisons between the two study locations for the different biological metrics (e.g., survival, weight, K, diet). Inter-site comparisons are confounded by temporal (i.e., seasonal) differences (primarily in temperature and hydrology) as well as differences in acclimation period at the hatchery and age of fish when placed in enclosures. However, we note that fish kept in enclosures at the DWSC site noticeably decreased in weight and K after the field deployment. One plausible reason for the difference is decreased food availability at the DWSC site compared to the RV site, as observed by the overall lower zooplankton biomass (Fig 6). Another possibility is that the DWSC site deployment overlapped with the natural period of gamete production based on water temperatures [51], which may focus energy on gamete production and away from somatic growth. Captive-reared Delta Smelt females tend to reduce their feed intake considerably as they near spawning [52]. Lastly, fish transferred from the FCCL to the DWSC site were acclimated at the FCCL for 35 days longer than the fish used at the RV site. The desire to use the same cohort of fish, space issues at the hatchery (i.e., no additional tanks available), and logistical infeasibility of conducting both deployments simultaneously constrained the experimental design in this regard. Since the acclimation in the hatchery included swimming under constant flow conditions to prepare them for flows in the Delta, fish going to the DWSC site were acclimated for over twice as long as RV fish. Starting off the study with fish that had experienced such conditions may have caused fish to lack sufficient energy to constantly forage for food, negatively influencing the change in weight for fish at the DWSC site.

No large differences in Delta Smelt responses were detected among the three enclosure types. We observed reduced survival for one enclosure (design B; 88% survival for one replicate compared to 100% survival for the other replicate) at Rio Vista (Table 3). The observed survival at Rio Vista generally matched the pattern expected from the idea that greater flow reductions would lead to greater survival, with highest observed survival in design A, followed by design C, then design B (Table 3). This pattern was not replicated at the DWSC site (Table 3), so it may be a result of higher water velocities at Rio Vista (Table 4). However, any survival differences were small, and the relationships are not significant. Additionally, we believe that these observed differences are subtle relative to the range of velocities experienced by wild Delta Smelt and do not represent a clear benefit of one enclosure design over another, given that survival was high across all designs.

There was no evidence of fish escape from any of the enclosure types at either of the deployment sites. We did not observe any evidence of tampering or damage that would have resulted in an opportunity for fish to escape. Furthermore, many of our enclosures had 100% recovery of fish (alive and dead) and for those enclosures where some fish were not recovered, the most parsimonious explanation is that missing fish sank and decomposed or were consumed by other organisms before we could recover them. Sexually staging a subset of fish resulted in no spent females, indicating a low probability that spawning inside the enclosures occurred (data not included).

Foraging success was significantly different across enclosure types at RV, with design B (larger mesh size) providing a modest improvement over the other two designs. This is likely due to larger-sized prey entering into the enclosures and being subsequently consumed by the Delta Smelt. We believe there is likely a tradeoff with the larger mesh between higher foraging success due to increased prey availability and risk of higher energetic demands or impingement on the enclosure due to increased velocities.

The observed diet composition data was consistent with observations from wild fish. Prior work has shown that Delta Smelt diet is largely comprised of amphipods, copepods, and cladocerans [53]. Delta Smelt dietary shifts occur based on developmental stage and food availability, including seasonal use of Cladocera [39,54,55]. In this study, comparisons should only be made between the last zooplankton tows (i.e., week 5 in Fig 6) and Delta Smelt diets for each deployment, since digestion would remove all traces of prey from earlier in the deployment from the gut contents [56]. Despite the ambient biomass difference being small at RV (Fig 6), Cladocera made up a substantially greater proportion of the diet across all three enclosure types (Fig 5). An even starker contrast is seen at the DWSC site. Cladocera contributed only a small amount to the zooplankton biomass (Fig 6) while being half of the prey consumed (Fig 5). The Cladocera species found in the diet contents (e.g., *Daphnia*) were large-bodied zooplankton, indicating that enclosure material did not preclude entry of larger prey items.

As noted earlier, the zooplankton biomass at RV was significantly higher than DWSC. One source contributing to increased lower trophic productivity in the northern Delta is the Yolo Bypass, a floodplain of the Sacramento River, known for having high productivity during flooding [57,58]. During the period of both deployments, there were inundation events in the Yolo Bypass that activated floodplain productivity [59]. As this productivity moved downstream from the floodplain, we would expect these resources to be more available to caged Delta Smelt at RV, a location directly downstream from the Yolo Bypass, compared with the DWSC, a location several kilometers up a dead-end channel from where the Yolo Bypass enters the Delta. This differential access to floodplain productivity is corroborated by both the taxonomic composition of ambient zooplankton sampling (Fig 6) and fish diets (Fig 5) at our study sites, which indicate a higher abundance of cladocerans, commonly found in floodplains, at RV [60].

Water quality metrics from both study locations generally reflected typical winter/spring conditions (cooler temperatures, increased turbidity, decreased salinity, etc.) in the Estuary during a wet year [61] and fell within ranges consistent with Delta Smelt catches in the field [22,62]. The DWSC site, while being a long, deep channel, is effectively a dead-end slough with higher residence time and flow that is entirely driven by the tides. This site also has large, ocean-going ship traffic that passed close by the cages, causing relatively large wakes. Based on remote camera footage, we know of at least two ships passing by the DWSC enclosures during our deployment, and the effect of this disturbance on the fish may have affected fish condition.

## Conclusions

While captive breeding commonly represents an important tool in the conservation of endangered fishes, the ability to culture fish in a hatchery setting is only an initial step in potential management activities such as supplementation [63]. However, there are numerous regulatory hurdles for the release of hatchery stocks into the wild (e.g., Section 7 consultation for listed species; [3,49]). For an environmentally sensitive pelagic species such as Delta Smelt, these issues are amplified due to challenges with transport and timing of release, compared to hardier species like Chinook Salmon. In this context, we developed novel field enclosures that enabled zooplankton to enter and subsequently be consumed by fish and supported the survival and growth of this imperiled species. Hence, our study represents an important initial step in research to inform supplementation actions for Delta Smelt and supports the potential use of captive-reared Delta Smelt as a surrogate for wild fish to evaluate other conservation-related actions such as habitat restoration and flow management [64]. While many questions remain about whether and how to acclimate fish for releases, along with how to choose locations and times for release, our enclosures provide a valuable tool to begin addressing some of these key knowledge gaps for successful supplementation. We believe that this type of tool will be helpful not only for Delta Smelt conservation, but also for other environmentally sensitive and endangered fishes.

## Supporting information

S1 Table Summary of summed prey abundance and percentage of total prey abundance by taxon at Rio Vista (RV) and Deepwater Shipping Channel (DWSC). (DOCX)

S2 Table Summary of total organism biomass and percentage of total site biomass at Rio Vista (RV) and Deepwater Shipping Channel (DWSC). (DOCX)
