# Supplementary material for: Captive-reared Delta Smelt (Hypomesus transpacificus) exhibit high survival in natural conditions using in situ enclosures
Source: PLoS One. 2023 May 26;18(5):e0286027. doi: 10.1371/journal.pone.0286027 (PMC10218733; doi:10.1371/journal.pone.0286027)
Supplement: S1 Table — (DOCX) [file pone.0286027.s001.docx]

| **Site** | **Taxonomic Grouping** | **Prey Taxa** | **Sum of Count** | **Percent of  Total Count** |
| --- | --- | --- | --- | --- |
| RVR | Cladocera | Daphnia sp. | 710 | 45.87 |
| RVR | Cyclopoida | Acanthocyclops sp. | 421 | 27.2 |
| RVR | Cladocera | Cladocera | 120 | 7.75 |
| RVR | Cladocera | Simocephalus sp. | 94 | 6.07 |
| RVR | Cyclopoida | Eucyclops sp. | 44 | 2.84 |
| RVR | Cladocera | Ceriodaphnia sp. | 37 | 2.39 |
| RVR | Cyclopoida | Diacyclops thomasi | 29 | 1.87 |
| RVR | Calanoida | Diaptomidae | 17 | 1.1 |
| RVR | Cladocera | Chydorus sp. | 15 | 0.97 |
| RVR | Calanoida | Osphranticum labronectum | 14 | 0.9 |
| RVR | Cyclopoida | Cyclopoida | 8 | 0.52 |
| RVR | Diptera | Chironomidae | 6 | 0.39 |
| RVR | Amphipoda | Crangonyx sp. | 4 | 0.26 |
| RVR | Cladocera | Sida sp. | 4 | 0.26 |
| RVR | Other | Insecta | 4 | 0.26 |
| RVR | Amphipoda | Hyallela azteca | 3 | 0.19 |
| RVR | Ostracoda | Ostracoda | 3 | 0.19 |
| RVR | Cladocera | Bosmina sp. | 2 | 0.13 |
| RVR | Amphipoda | Amphipoda | 1 | 0.06 |
| RVR | Calanoida | Calanoida | 1 | 0.06 |
| RVR | Calanoida | Eurytemora affinis | 1 | 0.06 |
| RVR | Cladocera | Chydoridae | 1 | 0.06 |
| RVR | Cladocera | Scapholeberis sp. | 1 | 0.06 |
| RVR | Cyclopoida | Macrocyclops sp. | 1 | 0.06 |
| RVR | Cyclopoida | Mesocyclops sp. | 1 | 0.06 |
| RVR | Cyclopoida | Tropocyclops sp. | 1 | 0.06 |
| RVR | Other | Acari | 1 | 0.06 |
| RVR | Other | Culicidae | 1 | 0.06 |
| RVR | Other | plant matter | 1 | 0.06 |
| DWSC | Cyclopoida | Acanthocyclops sp. | 2551 | 43.75 |
| DWSC | Cladocera | Chydorus sp. | 782 | 13.41 |
| DWSC | Cyclopoida | Cyclopoida | 731 | 12.54 |
| DWSC | Cladocera | Bosmina sp. | 622 | 10.67 |
| DWSC | Cyclopoida | Diacyclops thomasi | 278 | 4.77 |
| DWSC | Calanoida | Eurytemora affinis | 243 | 4.17 |
| DWSC | Calanoida | Calanoida | 202 | 3.46 |
| DWSC | Cladocera | Daphnia sp. | 122 | 2.09 |
| DWSC | Calanoida | Sinocalanus doerrii | 67 | 1.15 |
| DWSC | Calanoida | Diaptomidae | 40 | 0.69 |
| DWSC | Cladocera | Ceriodaphnia sp. | 39 | 0.67 |
| DWSC | Cyclopoida | Eucyclops sp. | 32 | 0.55 |
| DWSC | Cladocera | Simocephalus sp. | 28 | 0.48 |
| DWSC | Harpacticoda | Harpacticoida | 18 | 0.31 |
| DWSC | Diptera | Chironomidae | 12 | 0.21 |
| DWSC | Ostracoda | Ostracoda | 11 | 0.19 |
| DWSC | Cladocera | Cladocera | 9 | 0.15 |
| DWSC | Cladocera | Scapholeberis sp. | 9 | 0.15 |
| DWSC | Cladocera | Chydoridae | 8 | 0.14 |
| DWSC | Cyclopoida | Halicyclops sp. | 6 | 0.1 |
| DWSC | Other | Fish | 4 | 0.07 |
| DWSC | Other | Insecta | 3 | 0.05 |
| DWSC | Amphipoda | Americorophium sp. | 2 | 0.03 |
| DWSC | Calanoida | Pseudodiaptomus forbesi | 2 | 0.03 |
| DWSC | Cladocera | Eurycercus sp. | 2 | 0.03 |
| DWSC | Other | Acari | 2 | 0.03 |
| DWSC | Other | Hemiptera | 2 | 0.03 |
| DWSC | Amphipoda | Hyallela azteca | 1 | 0.02 |
| DWSC | Cladocera | Alona sp. | 1 | 0.02 |
| DWSC | Cladocera | Leydigia sp. | 1 | 0.02 |
| DWSC | Other | Bivalvia | 1 | 0.02 |
| DWSC | empty | empty | 0 | 0 |
