# Supplementary material for: Captive-reared Delta Smelt (Hypomesus transpacificus) exhibit high survival in natural conditions using in situ enclosures
Source: PLoS One. 2023 May 26;18(5):e0286027. doi: 10.1371/journal.pone.0286027 (PMC10218733; doi:10.1371/journal.pone.0286027)
Supplement: S2 Table — (DOCX) [file pone.0286027.s002.docx]

| **Taxa** | **Organism** | **Biomass at RV (µgC/m^3^)** | **Percent of Total RV Biomass** | **Biomass at DWSC (µgC/m^3^)** | **Percent of Total DWSC Biomass** |
| --- | --- | --- | --- | --- | --- |
| Cyclopoid | Acanthocyclops vernalis adult | 1241.9 | 3.9 | 31.9 | 0.3 |
| Cladocera | Alona | 173.4 | 0.6 | 13.3 | 0.1 |
| Cladocera | Alonella | 21.0 | 0.1 | 4.5 | 0.0 |
| Cladocera | Bosmina | 1284.8 | 4.1 | 255.2 | 2.1 |
| Calanoid | Calanoid copepodid | 700.0 | 2.2 | 2618.4 | 21.1 |
| Cladocera | Ceriodaphnia | 1361.6 | 4.3 | 90.4 | 0.7 |
| Cladocera | Chydorus | 1313.0 | 4.2 | 73.2 | 0.6 |
| Cyclopoid | Cyclopoid copepodid | 17577.4 | 55.8 | 6220.7 | 50.2 |
| Cladocera | Daphnia | 4715.2 | 15.0 | 226.2 | 1.8 |
| Cyclopoid | Diacyclops thomasi | 1016.9 | 3.2 | 121.2 | 1.0 |
| Cyclopoid | Eucyclops pectinifer | 44.9 | 0.1 | 23.1 | 0.2 |
| Cyclopoid | Eucyclops prionophorus | 54.6 | 0.2 | 0.0 | 0.0 |
| Calanoid | Eurytemora affinis adult | 41.7 | 0.1 | 226.7 | 1.8 |
| Cladocera | Graptoleberis | 6.8 | 0.0 | 0.0 | 0.0 |
| Harpacticoid | Harpacticoids | 330.2 | 1.0 | 115.3 | 0.9 |
| Calanoid | Hesperodiaptomus franciscanus | 0.0 | 0.0 | 11.3 | 0.1 |
| Cladocera | Kurzia | 42.1 | 0.1 | 3.3 | 0.0 |
| Cladocera | Leydigia | 94.2 | 0.3 | 2.3 | 0.0 |
| Cyclopoid | Limnoithona tetraspina | 0.0 | 0.0 | 164.1 | 1.3 |
| Cyclopoid | Macrocyclops albidus | 0.0 | 0.0 | 4.4 | 0.0 |
| Cyclopoid | Mesocyclops edax | 0.0 | 0.0 | 12.6 | 0.1 |
| Cyclopoid | Microcyclops rubellus | 640.4 | 2.0 | 9.4 | 0.1 |
| Cladocera | Monospilus | 11.2 | 0.0 | 12.6 | 0.1 |
| Cyclopoid | Paracyclops sp. | 0.0 | 0.0 | 9.1 | 0.1 |
| Cladocera | Pseudochydorus | 21.7 | 0.1 | 0.0 | 0.0 |
| Calanoid | Pseudodiaptomus forbesi adult | 196.2 | 0.6 | 37.8 | 0.3 |
| Cladocera | Simocephalus | 417.2 | 1.3 | 8.1 | 0.1 |
| Calanoid | Sinocalanus doerrii adult | 0.0 | 0.0 | 2088.6 | 16.9 |
| Calanoid | Skistodiaptomus pallidus | 204.0 | 0.6 | 0.0 | 0.0 |

Microzooplankton were not included in these totals.
